# A Data-Driven Approach for Interpretable and Efficient Predictive Modeling: A Case Study in SARS-CoV-2 Protease Inhibitor Discovery Through Feature Selection

**DOI:** 10.3390/ph19030498

**Published:** 2026-03-18

**Authors:** Branislav Stanković, Sang-Yong Oh, Dušan Ramljak

**Affiliations:** 1 Department for Nuclear and Plasma Physics, Vinča Institute of Nuclear Sciences—National Institute of the Republic of Serbia, University of Belgrade, P.O. Box 522, 11001 Belgrade, Serbia; branislav.stankovic@vin.bg.ac.rs; 2 School of Professional Graduate Studies at Great Valley, The Pennsylvania State University, Malvern, PA 19355, USA; szo5310@psu.edu

**Keywords:** QSAR, SARS-CoV-2, 3CL^pro^, feature selection, data driven decision making, interpretability

## Abstract

**Background/Objectives**: Feature selection approaches should satisfy all evaluation criteria required by state-of-the-art chemoinformatic models. Our aim is to develop a methodology that is robust, interpretable and computationally efficient. **Methods**: This study presents a robust methodology for developing highly interpretable and computationally efficient predictive models, with a specific application in the discovery of SARS-CoV-2 main protease inhibitors. We evaluated various descriptor selection procedures to identify a transparent and reproducible approach that provides actionable insights for data-driven decisions. The models were trained and tested using molecules from the CHEMBL database and further validated on an external set of compounds. **Results**: Our findings demonstrate that a recently proposed procedure, combining the FeatureWiz algorithm with stepwise feature selection, is the only approach that satisfies all evaluation criteria required by state-of-the-art chemoinformatic models. In particular, we found that models based on two-dimensional descriptors and Ordinary Least Squares regression achieved the best results. **Conclusions**: Our framework and the choices made offer significant advantages in a decision-making context due to their inherent interpretability and computational efficiency. Our derived models, benchmarked against those in the literature, serve as effective, transparent tools for the rapid and reliable prediction of biological activity, providing a validated framework for data-driven decisions in drug discovery and beyond.

## 1. Introduction

The SARS-CoV-2 pandemic has had a profound global impact, affecting almost 800 million people and causing more than 7 million deaths by November 2025 [1]. Even with the widespread use of vaccines, the ongoing emergence of new cases and deaths highlights the ongoing need for effective therapeutic drugs. Various pharmaceutical classes have been tested as drugs for COVID-19, including antibacterials, antimalarials, antivirals, immunomodulators, and others [2,3]. Concerns persist regarding vaccine safety for individuals with allergies, immune disorders, or those who are pregnant, and their efficacy against evolving coronavirus variants remains uncertain.

A primary target for pharmaceutical interventions is the coronavirus 3-chymotrypsin-like protease ($M^{\mathrm{pro}}$ or ${3\mathrm{CL}}^{\mathrm{pro}}$). This key protease, composed of approximately 306 amino acids, is essential for viral replication and transcription. Its substrate-recognition pocket is highly conserved across all coronaviruses and could help to find a drug that would work in the event of a new pandemic. It features key residues, including the nucleophilic sulfur of Cys 145 and the imidazole ring of His 41, which are crucial for its enzymatic activity. Therefore, disrupting the function of these residues is an effective strategy for inhibiting ${3\mathrm{CL}}^{\mathrm{pro}}$ [4,5].

Given the challenges and high costs of traditional drug discovery, computational approaches have become indispensable. Quantitative Structure–Activity Relationship (QSAR) modeling allows researchers to predict biological activity directly from molecular structure, enabling faster and more efficient identification of promising compounds. In drug repurposing, QSAR is particularly valuable as it can reveal new therapeutic potentials for existing molecules, thereby reducing the development time and the associated cost.

Data-driven methods leveraging machine learning (ML) have greatly enhanced the potential of QSAR modeling by enabling the efficient handling of large chemical datasets and the extraction of complex, non-linear patterns between structure and activity. This integration makes QSAR a powerful tool for predicting biological activity, guiding drug discovery, and supporting repurposing efforts. Using ML, QSAR can provide faster, more accurate, and generalizable insights compared to traditional approaches.

This study introduces a robust, highly interpretable, and computationally efficient methodology for developing predictive models in drug discovery, specifically demonstrated through the identification of potent SARS-CoV-2 main protease inhibitors.

Our primary contribution is the development and validation of a transparent and reproducible approach. We rigorously evaluated various descriptor selection techniques and demonstrated that a combined approach using the FeatureWiz algorithm followed by stepwise selection yields superior performance compared to the benchmarks from the literature. In addition to providing actionable insights for predictive models, it is the only approach that satisfies all evaluation criteria required by state-of-the-art chemoinformatic models.

We successfully trained and tested a model using molecules from the CHEMBL database, and externally validated the model using an external set of compounds. External set is added to ensure generalizability. Our key finding is that models built on two-dimensional molecular descriptors and the Ordinary Least Squares (OLS) regression method achieved the best overall results. These specific methodological choices constitute a significant contribution by providing inherent interpretability and high computational efficiency, which are critical factors for rapid, data-driven decisions in a drug discovery setting.

Ultimately, the models derived from this validated framework serve as effective, transparent tools for the rapid and reliable prediction of biological activity, establishing a crucial foundation for data-driven decisions in the fight against COVID-19 and accelerating other therapeutic programs. A key practical outcome of this work is the proposal to integrate these models into high-throughput screening (HTS) processes.

The remainder of the paper is organized as follows. In Section Interpretable and Efficient Predictive Modeling Background we explore the relevant prior research on interpretable and efficient predictive modeling. The results and their implications are discussed in Section 2. The dataset, design, and implementation of our approach are presented in Section 3. Finally, Section 4 provides concluding remarks and outlines directions for future research.

### Interpretable and Efficient Predictive Modeling Background

Quantitative structure–activity relationship modeling has long been recognized as a cornerstone of computational drug discovery, operating on the principle that the biological activity of a compound is fundamentally influenced by its chemical structure. This structural–activity paradigm has been widely applied to ${3\mathrm{CL}}^{\mathrm{pro}}$, a highly conserved viral enzyme that is essential for the SARS-CoV-2 replication, and therefore a critical target for therapeutic intervention [6,7,8,9,10,11,12,13,14,15,16]. Several QSAR studies on ${3\mathrm{CL}}^{\mathrm{pro}}$ inhibitors reveal that branching within molecular scaffolds generally reduces inhibitory activity [6,7,8]. Authors in [14] demonstrated that electron-donating substituents capable of hydrogen bonding enhance activity, while authors in [15] showed that the spatial distribution of electronegativity strengthens intermolecular polar interactions and minimizes intramolecular effects. Together, these findings underscore the importance of electronic and structural properties in shaping the inhibitory potential.

Heteroatoms, particularly nitrogen and sulfur, have been highlighted as key determinants of inhibitory potency in multiple studies. Authors in [6] reported that the pyridine and thiophene rings enhance activity, whereas imide and thioimide groups reduce it. Authors in [14] further identified the pyrrolidine and imidazole moieties as contributing to strong inhibition. Authors in [10] found that single nitrogen-containing rings, such as pyridine and piperidine, are favored over multi-heteroatom scaffolds such as pyridazine, pyrimidine, thiazole, and pyrazole. In contrast, secondary amides have been repeatedly associated with a decrease in inhibitory potential [7,8,10]. Beyond these structure–activity trends, authors in [16] incorporated quantum chemical descriptors such as the lowest unoccupied molecular orbital ($E_{\mathrm{LUMO}}$) and polarizability (α), which are well-established in enzyme–substrate interaction studies [17,18,19,20]. These results highlight the diversity of molecular features that can inform predictive modeling but also reveal the challenges of consistently selecting descriptors that balance predictive accuracy and mechanistic interpretability.

The selection of descriptors remains a pivotal step in QSAR modeling because descriptors serve as the quantitative link between chemical structure and biological activity. Traditional strategies—such as genetic algorithms, stepwise regression, and backward elimination—have been extensively used to identify informative descriptors [6,7,8,9,10,11,12,13,14,15,16]. However, these approaches can be sensitive to multicollinearity and data dimensionality, limiting their reproducibility. Recent work has proposed transparent, reproducible selection methods, including the integration of novel algorithms such as FeatureWiz with conventional stepwise procedures [21]. These techniques not only address limitations that previous models exhibited, but also emphasize interpretability, which is a priority in modern data-driven drug discovery where decision-making requires both reliability and clarity.

The modeling methodology is equally decisive in shaping the utility of QSAR frameworks. Although 3D descriptors and nonlinear learning algorithms can capture subtle structural effects, they often produce models that are computationally expensive and difficult to interpret for practitioners. By contrast, 2D descriptors provide a tractable balance between simplicity and informativeness, allowing for rapid, interpretable predictions that are more easily adopted in pharmaceutical pipelines [22]. Similarly, regression methods such as OLS remain highly valued for their transparency. Unlike complex black-box approaches, OLS coefficients directly map molecular features to activity, providing decision-makers with actionable insights into structural modifications. The computational efficiency of OLS further enhances its suitability for large-scale screening and real-time prioritization in drug discovery pipelines [23,24,25].

More broadly, the value of simplicity aligns with the principle of Occam’s Razor widely used in ML, advocating that models should be as simple as possible while still capturing essential patterns. Transparent and efficient models foster trust, reproducibility, and regulatory acceptance, which are essential for translational impact in fields like antiviral drug discovery. Indeed, several studies demonstrate that parsimonious models can achieve predictive performance comparable to or even exceeding that of more complex architectures [26,27,28]. Together, this literature underscores the urgent need for QSAR frameworks that integrate robust descriptor selection with transparent modeling, allowing actionable insights that are both statistically reliable and practically relevant to accelerate COVID-19 therapeutic development.

## 2. Results and Discussion

### 2.1. Evaluation of Models

**Figure 1:**
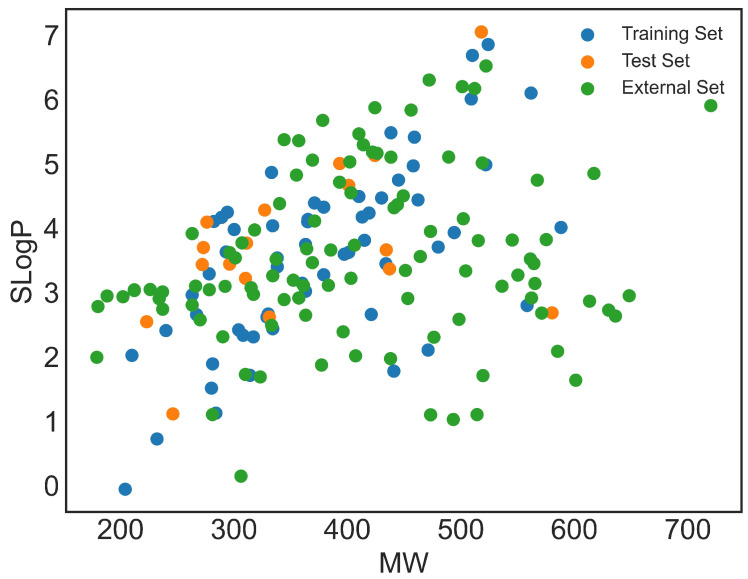
The plot of SlogP vs. MW for training (blue circles), test (orange circles), and external (green circles) set.

Assessing the predictive capacity of the developed models is based on understanding the distribution of molecules in the chemical space. The SlogP vs. MW space is a traditional representation widely used in cheminformatics to visualize the distribution of compounds in a simplified physicochemical space. It does not reflect the full structural space defined by the molecular descriptors used for model development. Thus, this representation is included only to illustrate how molecules are positioned in a commonly used chemical space. As shown in Figure 1, the molecules in the training, test, and external sets exhibit a diverse chemical profile. The training and test sets occupy similar regions, which is essential for consistent model applicability. The external dataset covers a slightly broader chemical space, confirming its suitability for validating the model’s ability to predict the activity of novel compounds.

### 2.2. Model Selection and Validation

In the context of data-driven decision-making, the choice of molecular descriptors is a critical first step. Although 3D descriptors can offer detailed information, their calculation is time-consuming and can be prone to inaccuracies, making them less suitable for rapid large-scale analysis. The majority of literature on QSAR models for ${3\mathrm{CL}}^{\mathrm{pro}}$ inhibitors focuses on 2D descriptors, which are simpler and more directly related to chemical constitution. The computational efficiency and greater interpretability of 2D descriptors are a significant advantage for decision-makers who need to quickly understand the structural features driving a prediction. For this reason, our study utilized 1613 2D molecular descriptors from the Mordred package version 1.2.0.

Quantum-chemical descriptors are inherently interpretable and can readily guide (pharmaceutical) chemists in rational compound design. Furthermore, although they are traditionally considered computationally expensive, recent advances in machine learning allow their accurate estimation at significantly reduced computational cost. Therefore, two quantum-chemical descriptors from a previous study of [16] were added to the 1613 2D molecular descriptors from the Mordred package version 1.2.0. From the initial set of descriptors, 969 descriptors remained after excluding those with missing or non-numerical values.

The choice of regression model plays a crucial role in data-driven decision-making. Although complex “black box” models can sometimes offer marginal improvements in predictive accuracy, interpretability often takes precedence when clear insights and transparency are essential. Models that reveal how descriptors influence activity are particularly valuable in guiding practical applications.

The case when OLS performs well suggests that a strong, simple linear relationship underlies the data, making the more interpretable model the preferred choice for this application. OLS coefficients directly indicate how each descriptor relates to activity, offering explainability by design. For example, a positive coefficient immediately signals that increasing the corresponding descriptor tends to increase predicted activity—providing drug designers with straightforward and actionable insights.

To generate the results, a genetic algorithm was applied to construct 500 models; however, none fulfilled the QUIK rule test or the selection criteria outlined in Section 3.5. Similarly, none of the five models developed using SequentialFeatureSelector, RFE, and SelectKBest algorithms passed the QUIK rule test, suggesting the presence of multicollinearity in all cases. In contrast, models obtained through RFE, SequentialFeatureSelector with forward selection, and SelectKBest with the f_regression function (Equations (A1), (A2) and (A3), respectively) satisfied all metric-based evaluation criteria (Table A1 and Table A2).

Our descriptor selection process demonstrated that the FeatureWiz algorithm followed by stepwise feature selection was the most effective method. This procedure not only streamlines the modeling process but also helps to mitigate multicollinearity, ensuring that the selected descriptors provide unique and meaningful insights. Neither $E_{\mathrm{LUMO}}$ nor α was selected, suggesting that these descriptors may only be effective for the specific class of molecules investigated by [16] or that they require a more precise, time-consuming method for calculation when applied to larger datasets.

We present three selected models. Model 1 (Equation (1)) and Model 2 (Equation (2)) were developed using a correlation threshold of 0.99, while Model 3 (Equation (3)) was based on a threshold of 0.85. In particular, Model 2 incorporates 10 of the 14 descriptors included in Model 1, raising questions regarding the influence of the descriptor count on model performance.

(1) $$\begin{array}{cc} {\mathrm{pIC}}_{50} & =2.22\cdot 10^{-2}\cdot \mathrm{AATS}7Z+1.47\cdot \mathrm{GATS}5c\;-1.85\cdot \mathrm{GATS}2\mathrm{are}-4.34\cdot 10^{-3}\cdot \mathrm{EState}\_\mathrm{VSA}4\\ \; & -2.72\cdot 10^{-2}\cdot \mathrm{SMR}\_\mathrm{VSA}6+1.18\cdot n10\mathrm{FaRing}\;+3.35\cdot 10^{-2}\cdot \mathrm{EState}\_\mathrm{VSA}9-0.11\cdot \mathrm{StsC}\\ \; & +0.31\cdot \mathrm{naHRing}-3.57\cdot 10^{-2}\cdot \mathrm{EOE}\_\mathrm{VSA}10+0.30\cdot \mathrm{NaaaC}-0.24\cdot \mathrm{NssssC}\\ \; & -4.13\cdot 10^{-2}\cdot \mathrm{SssNH}-1.57\cdot 10^{-3}\cdot \mathrm{PEOE}\_\mathrm{VSA}6+1.36 \end{array}$$

(2) $$\begin{array}{cc} {\mathrm{pIC}}_{50} & =2.40\cdot 10^{-2}\cdot \mathrm{AATS}7Z+1.50\cdot \mathrm{GATS}5c\;-1.83\cdot \mathrm{GATS}2\mathrm{are}\;-0.13\cdot \;\mathrm{StsC}\\ \; & -3.41\cdot 10^{-2}\cdot \mathrm{SMR}\_\mathrm{VSA}6+1.13\cdot n10\mathrm{FaRing}+3.33\cdot 10^{-2}\cdot \;\mathrm{EState}\_\mathrm{VSA}9\\ \; & +0.30\cdot \;\mathrm{naHRing}-3.81\cdot 10^{-2}\cdot \;\mathrm{PEOE}\_\mathrm{VSA}10+0.28\cdot \mathrm{NaaaC}+1.30 \end{array}$$

(3) $$\begin{array}{cc} {\mathrm{pIC}}_{50} & =0.48\cdot \mathrm{CIC}5+1.24\cdot \mathrm{AATS}0d+2.98\cdot \mathrm{GATS}2\mathrm{pe}-4.36\cdot 10^{-2}\cdot \mathrm{SMR}\_\mathrm{VSA}6\\ \; & +2.08\cdot \;\mathrm{GATS}5d+1.24\cdot n10\mathrm{FaRing}+2.46\cdot 10^{-2}\cdot \;\mathrm{EState}\_\mathrm{VSA}9\\ \; & -5.76\cdot 10^{-2}\cdot \mathrm{PEOE}\_\mathrm{VSA}10+0.32\cdot \mathrm{naHRing}\;+0.16\cdot \;\mathrm{SsBr}\;\\ \; & +0.45\cdot \mathrm{NsNH}2+0.32\cdot \;\mathrm{nBondsT}\;-0.42\cdot \;\mathrm{nG}12\mathrm{FAHRing}-3.8 \end{array}$$

The metrics for these models are summarized in Table 1 and Table 2. The consistently high *F*-values indicate that the selected descriptors collectively explain the variation in activity beyond chance. All models met the predefined criteria for robustness and predictability.

All models listed in Table 1 obey the QUIK rule, with no descriptors having a VIF higher than 5, indicating minimal multicollinearity. Moreover, the low values of coefficients of determination for the training set, $R_{\mathrm{Yscr}}^{2}$, and leave-one-out cross-validation, $Q_{\mathrm{Yscr}}^{2}$, calculated in Y-scrambling suggest the absence of chance correlation, further validating the reliability of the models for predicting inhibitory activity against the SARS-CoV-2 main protease.

The results demonstrate overall comparability across all models. Model 2 shows only slight variation compared to Model 1 (making it a good alternative), while Model 3 exhibits more significant differences, particularly in parameters related to prediction accuracy. These variations could impact model performance during external validation. It is worth noting that the models which we decided not to focus on and thus represented in the Appendix A by Equations (A1)–(A3) also exhibit slightly worse performance compared to Model 1 (Table A1 and Table A2 in the Experimental Section of Appendix A). Good performances in cross-validation experiments, as well as similar performances of the model when trained on the complete dataset, can indicate good stability of a model.

**Figure 2:**
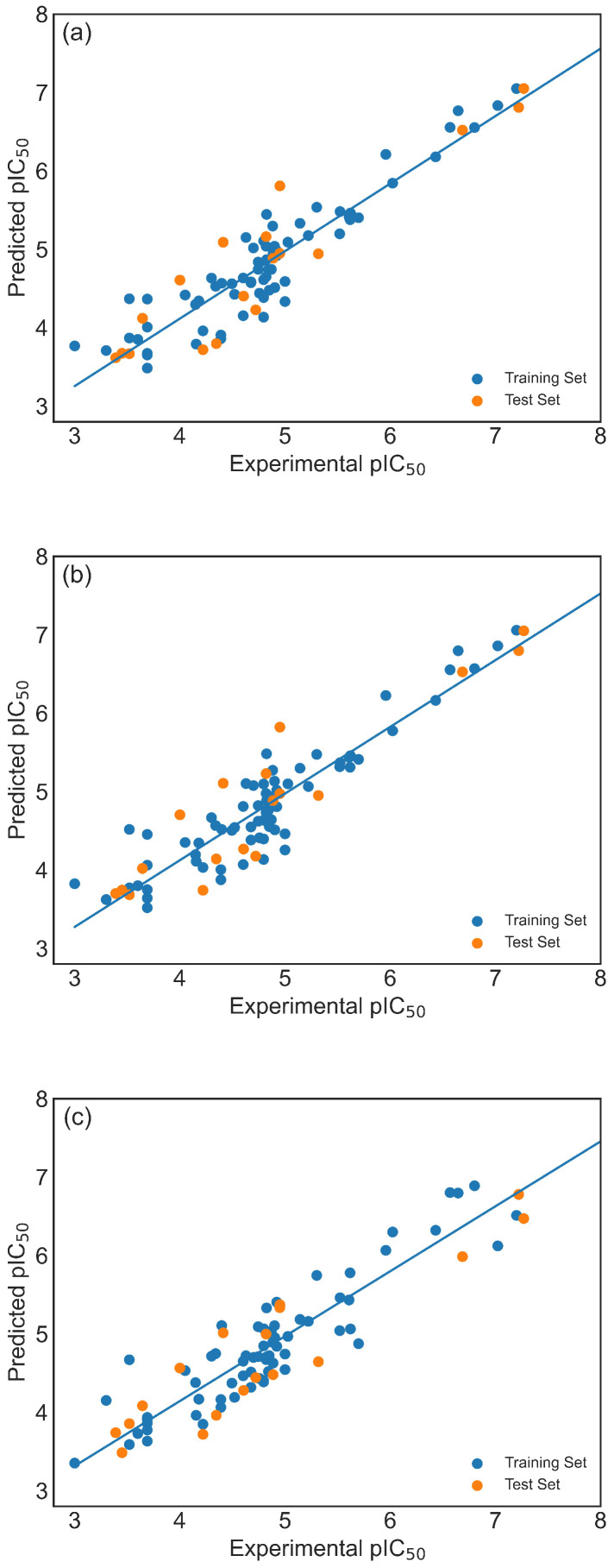
Scatter plot of predicted versus experimental ${\mathrm{pIC}}_{50}$ in the training (blue circles) and test (orange circles) set for: (**a**) Model 1, (**b**) Model 2, and (**c**) Model 3.

**Figure 3:**
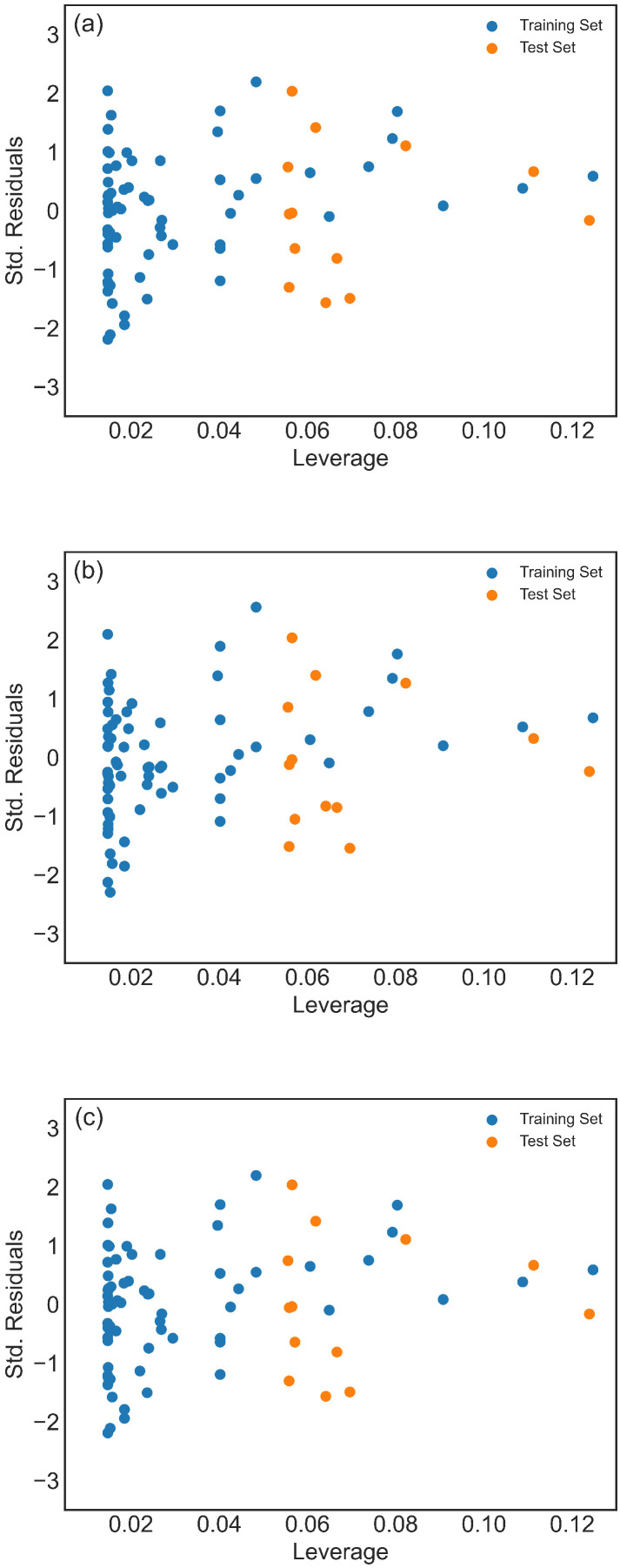
The Williams plot for training (blue circles) and test (orange circles) set: (**a**) Model 1 ($h^{*}=0.65$), (**b**) Model 2 ($h^{*}=0.48$), and (**c**) Model 3 ($h^{*}=0.61$).

To thoroughly investigate the performances of Models 1–3, scatter plots comparing experimental and predicted activity values for the training and test sets (Figure 2) were analyzed, along with the Williams plots (Figure 3). Williams plots are primarily designed to define and visualize the applicability domain of a QSAR/ML model, rather than to directly assess the quality of a train/test split. In principle, they may indicate a problematic split in cases where the training set compounds are clustered in a narrow region around the centroid of the descriptor space, while test set compounds occupy more extreme regions (i.e., show high leverage values). Since leverage reflects the distance from the centroid of the training set in descriptor space, a predominance of high-leverage test compounds could suggest structural imbalance and potential extrapolation. In our case, both training and test compounds occupy the same descriptor space region and are well within the defined applicability domain. The leverage values do not indicate structural extremity of the test compounds relative to the training set. Furthermore, the Williams plot is inherently constructed based on the selected training set and descriptor combination, which naturally centers the training compounds in the defined space. Taken together, the leverage analysis does not suggest structural imbalance between the training and test sets. This conclusion is further supported by the PCA analysis, which shows comparable distribution patterns for both sets in the descriptor space and will be presented separately. Thus, it is evident that all models demonstrate strong fitness and predictability. Models 1 and 2 show similar results, showing more accurate predictions for molecules with higher ${\mathrm{pIC}}_{50}$ values compared to Model 3 (Figure 2). Since one of the goals of the drug discovery process is to develop drugs with high ${\mathrm{pIC}}_{50}$ values, this represents a particular advantage.

Additionally, one molecule shows a slightly higher positive standard deviation (Figure 3c), although it does not surpass the critical value of 3 (i.e., no Y-outliers are detected). Moreover, all compounds in both the training and test set fall below the lowest threshold of $h^{*}=0.48$ (for Model 2, which has the lowest number of descriptors), indicating the absence of response outliers and suggesting that predictions of inhibitory activity against the SARS-CoV-2 main protease could be extrapolated by all three models (Figure 3).

To further ensure that the model does not have multicollinearity and overfitting issues and to confirm that all descriptors within the model are relevant, several other linear regression methods were performed. Even though other models are not strictly necessary to present a novel data-driven methodology for feature selection, their comparison with Models 1–3 strengthens its validation, as it demonstrates its advantages relative to alternative model configurations. Thus, we present Table A3 and Table A4 in the Experimental Section of Appendix A. As can be seen, performance decreases in the following order: OLS → Ridge → LassoLars → Bayesian Ridge → ARD → Lasso → Linear SVR. Since the OLS method unequivocally gives the best results and Model 1 and Model 2 show better performance than Model 3, in the remainder of the study, the focus of this research will be on the results obtained by Model 1 and Model 2. The fact that OLS performed optimally suggests that a strong, simple linear relationship underlies the data, making the more interpretable model the preferred choice for this application.

**Figure 4:**
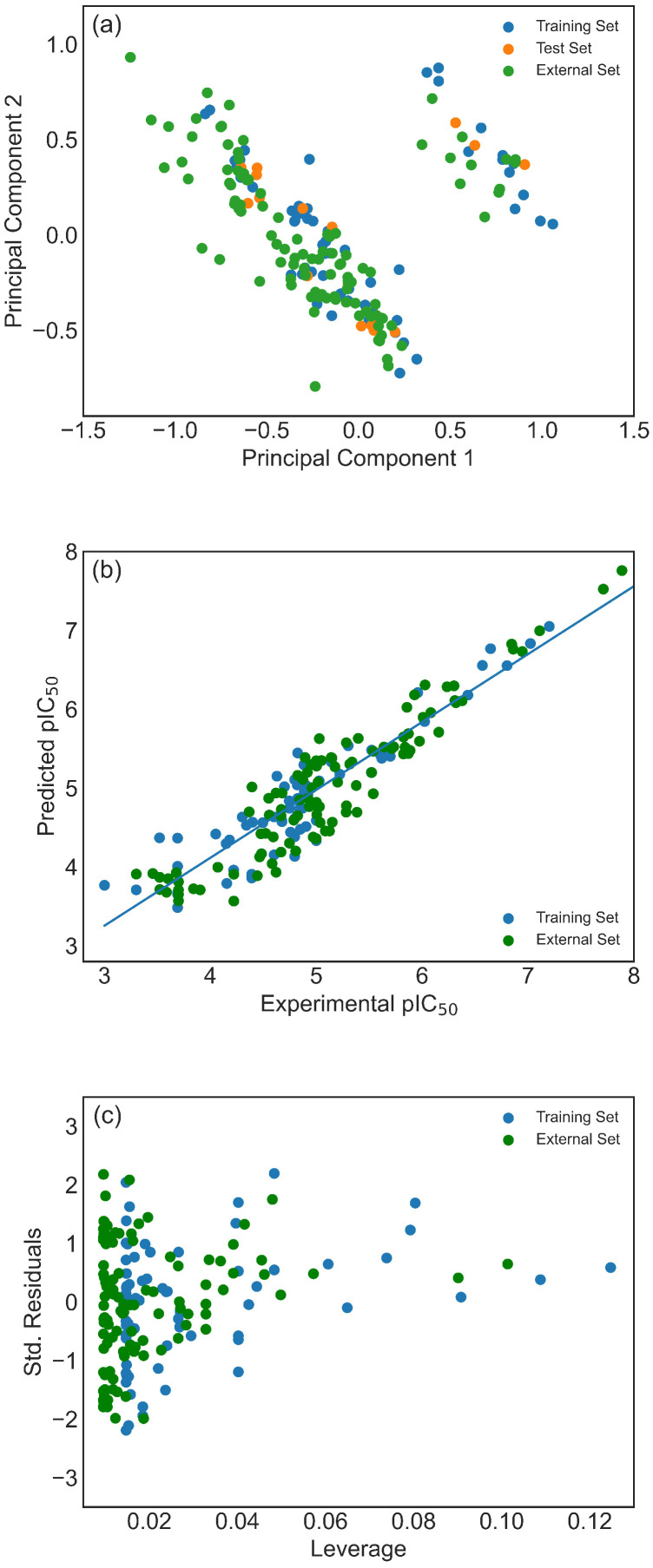
(**a**) The plot of the second vs. the first PC calculated on Model 1 descriptors for training, test, and external set, (**b**) scatter plot of predicted versus experimental ${\mathrm{pIC}}_{50}$ in the training and examination set, (**c**) the Williams plot for training and external set ($h^{*}=0.65$). (training set—blue circles, test set—orange circles, external set—green circles).

Given the relatively small size of the CHEMBL database dataset, questions may arise regarding the model’s capability to predict ${\mathrm{pIC}}_{50}$ values for compounds with more structurally diverse profiles. To address this, ${\mathrm{pIC}}_{50}$ values from various QSAR studies in the literature [6,7,8,9,10,11,12,13,14,15,16] were compiled to form an external dataset of molecules not present in the CHEMBL database. From the plot of the second versus the first principal component calculated on Model 1 descriptors, it can be observed that, similar to the previously discussed SlogP versus MW plot, molecules from the external dataset occupy a slightly broader chemical space than molecules from the CHEMBL database (Figure 4a), justifying its use. As a side note, it is convenient to notice that PCA divides the datasets into two distinctive subsets. The smaller subset consists of sulfur compounds and aromatic ketones, while the remaining molecules are in the larger subset.

**Figure 5:**
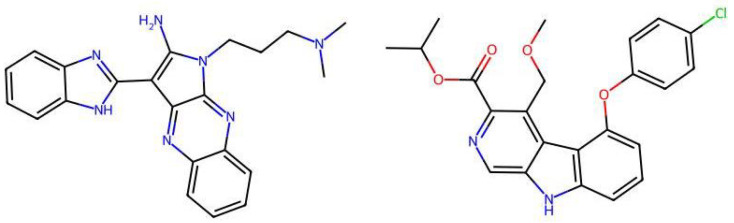
Two molecules form the external set that have higher ${\mathrm{pIC}}_{50}$ values than those found in the ChEMBL database.

The ability of Model 1 and Model 2 to predict ${\mathrm{pIC}}_{50}$ values of molecules from the external dataset was tested in two different ways: by using original models (results marked as 1e and 2e in Table 2) and using models trained on the full ChEMBL dataset (results marked as 1f and 2f in Table 2). As can be seen, all metrics show comparable values to those obtained on the internal test set. This is an excellent result given that, due to the heterogeneous sources of the external set chemicals, the higher variability in the results should be expected. In Figure 4b, a scatter plot comparing predicted versus experimental ${\mathrm{pIC}}_{50}$ values for the training and external sets shows that even the two molecules from the external set with higher ${\mathrm{pIC}}_{50}$ values than those in the training set are accurately predicted. Additionally, as observed from the Williams plot, no outliers are detected in the external set (Figure 4c). Therefore, there is a robust foundation to assert that the proposed model is suitable for predicting inhibitory activity within a broad chemical space (see Figure 5) and that consequently descriptor selection methodology can extract useful information from limited data. It is worth mentioning that, although Figure 4b,c present results of original Model 1, plots obtained by Model 2, as well as adequate models trained on the full ChEMBL dataset, do not differ significantly.

Due to the non-uniform distribution of data points, with a dense representation within the 4.5 to 5.5 range and sparse representation outside of it, the models’ ability to predict ${\mathrm{pIC}}_{50}$ values in these two sparse regions was tested. The models demonstrated similar performance to those presented in Table 1 and Table 2, confirming that they are not biased towards the majority range.

### 2.3. Comparison with Models Found in the Literature

To the best of our knowledge, Authors in [9] developed the only model existing in the literature using the dataset from the CHEMBL database. However, as shown in Table 3 detailing the performances of models found in the literature, this model is characterized by unsatisfactory statistics in external validation. Similarly, the models developed by [6,7,8,12,16] exhibit low values of $Q_{\mathrm{LOO}}^{2}$ and/or $Q_{F2}^{2}$. Furthermore, the model of [13] does not meet accuracy requirements due to a low value of $r_{m}^{2}$, while the model of [10] has this shortcoming along with a low value of ${\mathrm{CCC}}_{\mathrm{ext}}$ and a high value of $\Delta r_{m}^{2}$. Additionally, the model of [14] fails to meet the criterion $|R^{2}-Q_{\mathrm{LOO}}^{2}|\;<0.10$.

Therefore, among the models from the literature, only those by [11,15] are more closely aligned with the requirements of state-of-the-art QSAR modelling. However, ref. [15] did not calculate all relevant statistics to ensure that. Additionally, comparing these models directly with this study is challenging for several reasons. While 2D descriptors were employed in this study, the aforementioned research utilized 3D descriptors, which are not only more computationally intensive—requiring precise atom coordinates—but also often less intuitive. As discussed, this may result in less precise insights into the design of drug candidates compared to 2D descriptors. In addition, both models were developed for specific classes of inhibitors—ketone-based covalent inhibitors [11] and unsymmetrical aromatic disulfides [15]. Nevertheless, since the methodology presented in this study demonstrates comparable internal fitting parameters and superior external validation performance compared to that of [15], it can be inferred that it is better suited to predict the inhibitory activity of novel compounds. On the other side, while the model of [11] demonstrates better performance in 6 out of 9 parameters as shown in Table 3, once again, it is important to note that their model is specifically trained and tested on 29 derivatives of a certain compound, making its applicability limited to a narrow chemical space.

### 2.4. Mechanistic Interpretation

Mechanistic interpretation of QSAR models is an essential part of modelling because it provides insights into the biological or chemical processes underlying compound activity, thus enhancing scientific understanding and model validation. In other words, it ensures the model’s predictions are grounded in known principles, improving predictive power and reliability. In drug design, mechanistic insights guide the creation of effective and safe molecules, while also meeting regulatory requirements [29]. To better understand the mechanisms interpretation of Models 1 and 2, Table 4 provides the physical meaning of the corresponding descriptors. Additionally, Table 5 shows six molecules from the CHEMBL dataset (three with low ${\mathrm{pIC}}_{50}$ values and three with high ${\mathrm{pIC}}_{50}$ values), along with the values of all descriptors. This comparison highlights the differences in descriptor values between molecules with varying activity levels, further clarifying the relationship between molecular structure and inhibitory activity.

By reflecting its sensitivity to differences in atomic numbers among atoms at a lag of 7, the AATS7Z descriptor shows a positive correlation with activity. It favours molecules containing heavy atoms (such as Br, S, I, and Cl) and aromatic rings. These features can increase inhibitor activity by enhancing hydrophobicity and polarizability, which promotes intermolecular interactions over intramolecular interactions. EState_VSA9 descriptor is part of the EState family of descriptors and serves to quantify a specific aspect of Van der Waals surface area contributions based on electrotopological state considerations. In analysed datasets, molecules with high values of EState_VSA9 typically contain chlorine atoms (Table 5), which supports the frequent use of chlorine and other halogens as substituents in drugs, enhancing ligand–protein interactions via halogen bonds [30].

Molecules with higher values of GATS5c exhibit charge distribution over longer distances, allowing for more efficient interactions with amino residues. In contrast, molecules with lower values of GATS5c have a dense distribution of charged atoms (Table 5), resulting in lower hydrophobicity and reduced activity. Since oxygen and nitrogen atoms tend to have relatively low Gasteiger charges, molecules containing keto and ether groups, as well as nitrogen-containing rings, are favoured. Conversely, the last two molecules listed in Table 5 contain two nitrogen atoms from a pyrimidine ring at a topological distance of 5 from nitrogen in a secondary amide group, resulting in a low value for this descriptor. GATS5c was also identified in a study on 2,5-disubstituted furans as antimalarial drugs [31]. This adds to its importance, given that antimalarials have demonstrated in vitro efficacy against SARS-CoV-2.

The GATS2 descriptor has small values when nitrogen, sulfur, and oxygen atoms are at a topological distance of 2, i.e., for molecules containing (thio)amide groups and multiple heteroatom-containing rings. Similarly, the SssNH descriptor summarizes electrotopological states of nitrogen atoms in the form of –NH–, thus having higher values for secondary amides, which are known to exhibit low inhibitor activity [7,8,10].

Selection of n10FaRing is not surprising, given that molecules with large surface area, hydrophobic terminal groups, and stable aromatic substituents often show higher activities [9,11]. NaaaC is included in the model developed by [13], as well as in research on drugs with inhibitor activity against Alzheimer’s disease [32]. This descriptor has been associated with various interactions such as H-bonding, salt bridges, alkyl groups, and π-sigma, π-cation, and π-alkyl interactions.

naHRing shows a weak positive correlation with ${\mathrm{pIC}}_{50}$ values. While this might be surprising for any descriptor with integer values, naHRing has demonstrated particular significance in studies of activin receptor type-5 kinase inhibitors [33] and cannot be omitted from Models 1 and 2 without significantly affecting performance. Additionally, the majority of research highlights the significance of heteroatomic rings [6,10,14].

The descriptor StsC is related to the existence of a carbon atom with one triple and one single bond, i.e., cyano groups. According to findings by [14], inhibitor activity increases with the electron-donating ability of substituents. Therefore, since the cyano group is electron-withdrawing, the negative sign of the coefficient for StsC can be easily understood. Furthermore, NssssC has been selected, representing the number of quaternary carbon atoms. As said, it is known that branching decreases inhibitory activity [6,7,8]. In addition, this descriptor is included in the study of [8].

The PEOE_VSA10 descriptor, part of the partial equalization of orbital electronegativity descriptors, quantifies the Van der Waals surface area contribution from specific molecular segments. In other words, this descriptor reflects Van der Waals interactions associated with the partial charges of certain atoms or functional groups within the molecule. In analysed datasets, molecules featuring pyrimidine, thiazole, and pyrazole rings exhibit high PEOE_VSA10 values (Table 5). As said, the literature suggests that molecules with multiple heteroatom-containing rings tend to show lower inhibitory activity [10]. Notably, PEOE_VSA10 was selected in the study of HIV-1 protease inhibitors [34], some of which, like lopinavir/ritonavir, were proposed for SARS-CoV-2 treatment [2,3]. Similarly, the PEOE_VSA6 descriptor shows high values in molecules containing pyrimidine rings, secondary amides, and aromatic ketones. As mentioned, secondary amides are known for their lower activity, while aromatic ketones’ low inhibitory activity may be due to their keto groups’ poor electron donation capability. Additionally, the EState_VSA4 and SMR_VSA6 descriptors are selected, representing the electrotopological state and molar refractivity contribution of specific atoms or functional groups to the Van der Waals surface area. In analysed datasets, high EState_VSA4 values are observed in molecules containing pyrimidine rings and secondary amides, while SMR_VSA6 values peak when a S atom is bonded to the C2 atom of a pyrimidine ring and in molecules with chlorine. Given the similarity of molecules with high values of these descriptors, a detailed analysis is warranted to understand better their nuances (which is currently out of the scope of this study). However, PEOE_VSA6 and EState_VSA4 are only part of Model 1, which, as discussed, exhibits slightly better performance than Model 2, highlighting that these descriptors might be affecting the performance of molecular activity prediction.

A closer analysis of the dataset and the mechanistic interpretation of descriptors suggest that molecules with high inhibitory activity against SARS-CoV-2 main protease generally lack cyano and secondary amide groups, as well as quaternary carbons (i.e., StsC, NssssC, and SssNH descriptors have values 0). Eight of the top ten most active molecules exhibit relatively high AATS7Z values (associated with heavy atoms), and four of the top five most potent inhibitors contain chlorine, contributing to high AATS7Z and EState_VSA9 values. To the best of our knowledge, our approach resulted in models that are the first QSAR models to highlight these significant features. Additionally, the models favor aromatic rings (as represented by descriptors like n10FaRing, naHRing, and NaaaC), especially those with heteroatoms. However, this is under specific constraints, particularly from descriptors such as GATS5c and GATS2are, along with PEOE_VSA10, PEOE_VSA6, SMR_VSA6, and EState_VSA4. In summary, molecules featuring larger aromatic structures, chlorine or other heavy atoms, and multiple nitrogen-containing rings appear to be prominent inhibitors. Since each highly active molecule in the dataset can be categorized by these characteristics, combining these structural elements may lead to the synthesis of even more effective SARS-CoV-2 main protease inhibitors in the future.

### 2.5. Generalizability and Impact

The findings of this study, particularly the success of 2D descriptors and OLS regression, have broad implications for data-driven decision-making beyond QSAR. In any field dealing with high-dimensional data, the trade-off between model complexity and interpretability is a crucial consideration. Our work demonstrates that simpler, more interpretable models can not only perform comparably to complex ones, but can also be more valuable in practice by providing clear, actionable insights to decision-makers. By providing a robust and reproducible methodology for feature selection that empowers such models, this study offers a blueprint for building transparent, efficient, and trustworthy predictive systems in a variety of domains.

## 3. Materials and Methods

### 3.1. Datasets

Chemical structures exhibiting inhibitory activity against ${3\mathrm{CL}}^{\mathrm{pro}}$, used to train and test models, were sourced from the CHEMBL database (target ID = CHEMBL3927) [35]. To the best of our knowledge there is no explicit use of S9 metabolic activation in the original assays in the ChEMBL data. The half maximal inhibitory concentration (${\mathrm{IC}}_{50}$) values of all 86 molecules were converted to ${\mathrm{pIC}}_{50}$ values using the equation following

(4) $$\begin{array}{c} {\mathrm{pIC}}_{50}=\left(9-\log{\mathrm{IC}}_{50}\right) \end{array}$$

and utilized as target values (factor 9 was taken since ${\mathrm{IC}}_{50}$ are given in nM). The list of molecules used, together with their activities, is presented in Supporting Information File S1. Given the dataset’s wide range of activities spanning more than 4 log units, including molecules with activity at the nano-molar level, it can be considered suitable for developing QSAR models aimed at facilitating the discovery of novel drugs. Additionally, 105 molecules were gathered from the literature [6,7,8,9,10,11,12,13,14,15,16] and used as an external dataset both have higher heterogeneity and test the applicability of the models to predict the ${\mathrm{pIC}}_{50}$ values of novel compounds (Supporting Information File S2).

Molecular structures, initially represented as SMILES strings, were processed using RDKit version 2023.9.5 for SMILES parsing, salt removal (retaining the largest organic fragment), functional group normalization, and stereochemistry assignment. Duplicate entries were removed when multiple ${\mathrm{IC}}_{50}$ values were not reported for the same compound. Open Babel version 3.1.1 was employed for tautomer standardization, protonation state adjustment to physiological pH (7.40), and generation of three-dimensional structures. All molecules were visually inspected, and the results of tautomer standardization and protonation state assignment were verified using MarvinSketch version 24.3.2, which applies ChemAxon’s standardization engine. Conformational sampling was performed within Open Babel version 3.1.1 using the MMFF94 force field, with up to 50 conformations generated per molecule. Energy minimization was applied, and the lowest-energy conformer was selected for further analysis.

### 3.2. Molecular Descriptors

In this study, the Mordred 2D molecular descriptors [36] and two quantum-chemical descriptors from [16] were utilized. Hydrogens were treated as implicit, by default, in the calculation of Mordred 2D descriptors. The structure was optimized at $B3\mathrm{LYP}/6-31+G^{**}+\mathrm{LANL}2\mathrm{DZ}$ (on Br and I atoms) level of theory, at which quantum-chemical descriptors, $E_{\mathrm{LUMO}}$ and α, were also obtained. Vibrational frequency analysis was conducted to ensure the identification of minima on potential surfaces. DFT calculations were carried out using the polarizable continuum solvation model, with water as the solvent.

### 3.3. Selection of Descriptors and Modeling

**Figure 6:**
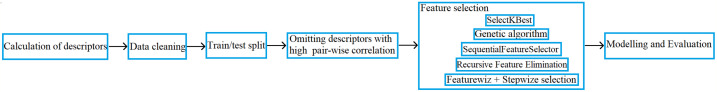
A schematic representation of the model development process.

A schematic representation of the model development process is provided in Figure 6. Descriptors containing missing values or non-numerical entries were omitted from the dataset in the data cleaning step. The data were then divided into training (80%) and test sets (20%) using the Kennard–Stone algorithm and normalized using the MinMaxScaler. The Kennard–Stone algorithm provides uniform and representative coverage of the descriptor space by selecting samples that are maximally spread across the chemical space, thereby reducing the risk of extrapolation within the applicability domain. It is deterministic and fully reproducible, ensuring the same split is obtained for a given dataset and distance metric. Moreover, it is particularly suitable for small to medium-sized datasets where random splitting may lead to unbalanced or poorly representative divisions. The MinMaxScaler was fitted exclusively on the training set. Obtained scaling parameters were subsequently applied to the test and external sets to prevent data leakage. Additionally, descriptors with a standard deviation less than 0.05 in the training set were excluded from the analysis. The final descriptor selection process was carried out using two different approaches.

In the initial step of the first approach, the set of descriptors was filtered to exclude any pairs exhibiting a pairwise correlation higher than 0.95. Specifically, descriptors with higher cumulative correlation coefficients were removed from consideration. Subsequently, a genetic algorithm (GA), as well as SequentialFeatureSelector, Recursive Feature Elimination (RFE), and SelectKBest algorithms (available in the sklearn module) were employed. In the case of GA, specific parameters were: population size = 100, mutation rate = 20, and number of generations = 500. SequentialFeatureSelector was used in both backward and forward directions, while SelectKBest was applied with two scoring functions, mutual_info_regression, and f_regression. The remaining parameters were kept at default values.

For the second approach, the FeatureWiz algorithm was applied, followed by stepwise feature selection following the approach applied in [21]. The FeatureWiz (version 0.5.7) configuration was as default except for corr_limit. The impact of different thresholds for acceptable pairwise correlations between descriptors was also explored. corr_limit values of 0.85 and 0.99 were set to assess their influence on the selection process.

Coefficients of determination in leave-one-out cross-validation, $Q_{\mathrm{LOO}}^{2}$, calculated for OLS regression, were used as the fitness function in both approaches. The ratio between the training set size and the number of descriptors was maintained at a minimum of 5. In other words, modelling was conducted using up to 14 descriptors.

Various linear regression models were employed for modelling: OLS, Ridge, Lasso (Least Absolute Shrinkage and Selection Operator), LassoLars (Lasso fitted with Least Angle Regression), Bayesian Ridge, ARD (Automatic Relevance Determination), and Linear SVR (Linear Support Vector Regression). Hyperparameters alpha (in the case of Ridge, Lasso, and LassoLars), alpha1, alpha2, lambda1, and lambda2 (in the case of Bayesian Ridge and ARD), and C (in the case of Linear SVR) were tuned in 5-fold cross-validation. The remaining parameters were kept at default values.

### 3.4. Methods for Inspection of Chemical Space

To delineate the applicability domain (AD), the leverage approach was employed, utilizing the Williams plot, which juxtaposes standard residuals against leverage (*h*). The *h* was calculated using the “hat” matrix formula $h=(x_{i}^{T}{\left(X^{T}X\right)}^{-1}x_{i})$, incorporating the training set descriptor matrix (*X*) and descriptor vector ($x_{i}$) for each molecule. A leverage threshold ($h^{*}$) was then established based on the number of descriptors (*p*) and the size of the training set (*n*), identifying molecules with $h>h^{*}$ as X-outliers. The value of $h^{*}=3\left(p+1\right)/n$ was used. Additionally, molecules surpassing a standardized residual critical value, typically set at 3, were deemed Y-outliers [37]. Furthermore, principal component analysis (PCA) was utilized to gain deeper insights into chemical space. Additionally, a plot of molecular weight (MW) against the Wildman-Crippen partition coefficient (SlogP) was employed to explore chemical space further.

### 3.5. Evaluation of Models

Model evaluation entailed a comprehensive examination, employing a diverse array of commonly utilized statistical metrics as outlined in the literature [23,38] and applied in [21], including root mean square error for the training set (${\mathrm{RMSE}}_{\mathrm{tr}}$), leave-one-out cross-validation (${\mathrm{RMSE}}_{\mathrm{cv}}$,) and test set (${\mathrm{RMSE}}_{\mathrm{ext}}$), adjusted coefficients of determination ($R_{\mathrm{adj}}^{2}$), coefficients of determination for the training set ($R^{2}$), leave-one-out ($Q_{\mathrm{LOO}}^{2}$), and leave-many-out ($Q_{\mathrm{LMO}}^{2}$) cross-validation, as well as three metrics derived from coefficients of determination $Q_{F1}^{2}$, $Q_{F2}^{2}$, and $Q_{F3}^{2}$. These metrics quantify predictive performance on the test set using different normalizations of the prediction error relative to the training set mean $Q_{F1}^{2}$, test set mean $Q_{F2}^{2}$, or variance scaling $Q_{F3}^{2}$, respectively. Moreover, concordance correlation coefficient in the training set (${\mathrm{CCC}}_{\mathrm{tr}}$), leave-one-out validation (${\mathrm{CCC}}_{\mathrm{cv}}$), and the test set (${\mathrm{CCC}}_{\mathrm{ext}}$), *F*-value, mean absolute error (${\mathrm{MAE}}_{\mathrm{ext}}$) in the test set, slopes of regression lines through the origin of predicted vs. experimental values (*k*), and experimental vs. predicted values ($k^{\prime }$) were evaluated, along with $r_{m}^{\prime 2},\;\Delta r_{m}^{2}$, and $R_{\mathrm{ext}}^{2},\left(R_{\mathrm{ext}}^{2}-R_{0}^{\prime 2}\right)/R_{\mathrm{ext}}^{2}$, and $\left(R_{\mathrm{ext}}^{2}-R_{0}^{\prime 2}\right)/R_{\mathrm{ext}}^{2}$. Acceptable models were considered those that meet the following criteria: (1) $R^{2},Q_{\mathrm{Fn}}^{2},Q_{\mathrm{LOO}}^{2}>0.70$, (2) $R_{\mathrm{ext}}^{2}>0.5$, (3) $|R_{0}^{2}-R_{0}^{\prime 2}|<0.30$, (4) $\left(\left(R_{\mathrm{ext}}^{2}-R_{0}^{\prime 2}\right)\right)/\left(R_{\mathrm{ext}}^{2}\right),\left(\left(R_{\mathrm{ext}}^{2}-R_{0}^{\prime 2}\right)\right)/\left(R_{\mathrm{ext}}^{2}\right)<0.10$, (5) $0.85<k,k^{\prime }<1.15$, (6) $|R^{2}-Q_{\mathrm{LOO}}^{2}|\;<0.10$, (7) $r_{m}^{2}>0.65$, (8) $\Delta r_{m}^{2}<0.20$, and (9) ${\mathrm{CCC}}_{\mathrm{ext}}\geq 0.85$ [39].

To address multicollinearity and chance correlation, a three-fold selection process was implemented. Initially, the QUIK (Q Under Influence of K) rule was utilized [40]. Following literature recommendations, only models with a difference of over 0.05 between two K indices were retained [29]. Subsequently, the Variance Inflation Factor (VIF) was computed, and models with each descriptor having a VIF value below 5 were passed to final test [41]. Y-Scrambling was then employed 200 times to ensure the robustness of the selected models.

### 3.6. Selection of the Best Model

The optimal model selection process utilized Multi-Criteria Decision Making (MCDM), more precisely the weighted sum model, integrating all measures of goodness-of-fit, robustness, and predictivity from the previous subsection to rank models. Additionally, the number of descriptors was factored in, ensuring a balanced assessment of model performance and simplicity. This dual approach ensures comprehensive evaluation, capturing both overall model performance via MCDM and the model’s complexity based on descriptor count.

### 3.7. Software

The modelling process was conducted using Python (v3.10.12) with standard libraries including NumPy (v1.25.2), pandas (v1.5.3), sklearn (v1.2.2), and statsmodels (v0.14.1), while visualization was facilitated by seaborn module (v0.13.1). Quantum-chemical descriptors were computed using the Gaussian 16 package [42]. Molecular descriptors were generated using the Mordred package (v1.2.0) [36]. Feature selection was executed utilizing the Featurewiz (v0.5.7) [43], genetic_selection (v0.6.0) [44], and sklearn modules. Preparation of data was done by RDKit 2023.9.5 [45], OpenBabel 3.1.1 [46], and MarvinSketch 24.3.2 [47].

## 4. Conclusions

In this study, we presented a robust QSAR modeling approach for identifying SARS-CoV-2 main protease inhibitors, emphasizing the value of interpretability and efficiency for data-driven decision-making. Our methodology, which combines the FeatureWiz algorithm with stepwise feature selection, successfully identified a set of 2D molecular descriptors that yield highly predictive models. The superior performance of OLS regression further underscores the importance of simple, transparent models for providing actionable insights. By demonstrating that a simpler approach can outperform more complex methods, we have shown that interpretability and computational efficiency are not merely desirable features but can be key drivers of a model’s success. The models presented here are not just predictive tools for QSAR; they represent a validated framework for data-driven decision-making that can be generalized to other fields where transparency and efficiency are critical for informed action.

To further advance this work and broaden its impact, we propose three distinct avenues for future research. First, exploration of advanced feature engineering. The current work utilized readily available two-dimensional descriptors. Future research should investigate the impact of incorporating more sophisticated feature engineering techniques, such as graph-based representations or molecular fingerprints derived from deep learning autoencoders. This will assess whether incremental gains in predictive power can be achieved without sacrificing the interpretability essential for drug discovery. Second, generalizability across therapeutic targets. The validated framework should be applied to a wider range of therapeutic targets beyond the SARS-CoV-2 main protease. Future studies will focus on testing the methodology’s generalizability and robustness against structurally diverse protein families and therapeutic areas to establish it as a universal standard for fast, interpretable QSAR modeling. Finally, third avenue would be the integration with High-Throughput Screening (HTS) pipelines and is the key practical outcome of this work. To maximize practical impact, the final models should be integrated into an automated, high-throughput computational screening pipeline. This will involve developing a user-friendly software module that can rapidly process large virtual libraries, provide immediate activity predictions, and flag compounds with optimal predicted properties and low synthetic complexity, thereby directly streamlining hit identification for experimental validation.

## Figures and Tables

**Table 1:** Internal statistical fitting parameters of the predictive QSAR models.

Model	$R^{2}$	$R_{\mathrm{adj}}^{2}$	${\mathrm{RMSE}}_{\mathrm{tr}}$	${\mathrm{CCC}}_{\mathrm{tr}}$	F	$Q_{\mathrm{LOO}}^{2}$	${\mathrm{RMSE}}_{\mathrm{cv}}$	${\mathrm{CCC}}_{\mathrm{cv}}$	$Q_{\mathrm{LMO}}^{2}$	$R_{\mathrm{Yscr}}^{2}$	$Q_{\mathrm{Yscr}}^{2}$
1	0.871	0.825	0.323	0.929	23	0.821	0.396	0.888	0.735	0.196	−0.371
2	0.865	0.824	0.335	0.919	32	0.797	0.409	0.884	0.714	0.142	−0.277
3	0.838	0.787	0.359	0.906	20	0.746	0.436	0.865	0.709	0.180	−0.379

**Table 2:** Predictive performance parameters of the selected QSAR models on the external test set.

Model	$R_{\mathrm{ext}}^{2}$	${\mathrm{RMSE}}_{\mathrm{ext}}$	$Q_{F1}^{2}$	$Q_{F2}^{2}$	$Q_{F3}^{2}$	${\mathrm{CCC}}_{\mathrm{ext}}$	${\mathrm{MAE}}_{\mathrm{ext}}$	$r_{m}^{2}$	$\Delta r_{m}^{2}$	k	$k^{\prime }$	$\frac{R_{\mathrm{ext}}^{2}-R_{0}^{2}}{R_{\mathrm{ext}}^{2}}$	$\frac{R_{\mathrm{ext}}^{2}-R_{0}^{\prime 2}}{R_{\mathrm{ext}}^{2}}$
1	0.865	0.425	0.865	0.864	0.759	0.927	0.360	0.791	0.124	0.993	0.999	0.000	0.029
2	0.860	0.430	0.862	0.861	0.754	0.914	0.366	0.772	0.128	0.989	1.004	0.001	0.038
3	0.832	0.471	0.834	0.834	0.705	0.899	0.435	0.679	0.162	1.019	0.973	0.014	0.098
1e	0.872	0.338	0.870	0.853	0.848	0.928	0.280	0.816	0.000	1.018	0.979	0.005	0.005
2e	0.859	0.337	0.870	0.858	0.848	0.925	0.274	0.796	0.077	1.000	0.995	0.001	0.016
1f	0.878	0.325	0.880	0.869	0.880	0.932	0.275	0.823	0.000	1.015	0.098	0.004	0.004
2f	0.861	0.334	0.871	0.859	0.853	0.926	0.272	0.796	0.075	1.004	0.990	0.000	0.010

**Table 3:** Comparison of the statistical performance of Models 1 and 2 with existing Quantitative Structure–Activity Relationship (QSAR) models from the literature.

Reference	Size	$R^{2}$	$R_{\mathrm{adj}}^{2}$	${\mathrm{CCC}}_{\mathrm{tr}}$	$Q_{\mathrm{LOO}}^{2}$	$R_{\mathrm{ext}}^{2}$	$Q_{F1}^{2}$	$Q_{F2}^{2}$	$Q_{F3}^{2}$	${\mathrm{CCC}}_{\mathrm{ext}}$	$r_{m}^{2}$	$\Delta r_{m}^{2}$
Model 1	86	0.871	0.825	0.925	0.821	0.865	0.865	0.864	0.759	0.927	0.791	0.124
Model 2	86	0.865	0.824	0.919	0.797	0.860	0.862	0.861	0.754	0.914	0.772	0.128
[6]	69	0.764	/	/	0.627	/	0.727	0.652	/	/	0.610	0.110
[7]	34	0.748	0.700	/	0.628	/	/	0.723	/	/	0.558	/
[8]	69	0.740	0.711	/	0.674	/	/	0.654	/	/	0.502	/
[9]	73	0.907	0.890	/	0.866	0.609	/	0.517	/	/	0.817	0.050
[10]	104	0.756	0.739	/	0.708	/	0.752	0.752	/	0.841	0.573	0.214
[11]	29	0.920	/	0.920	0.650	0.930	0.890	0.890	0.770	0.910	0.790	0.090
[12]	70	0.720	/	/	0.650	/	/	0.640	/	/	0.630	0.007
[13]	84	0.759	0.742	/	0.705	0.870	0.860	0.859	/	/	0.595	0.182
[14]	48	0.955	/	0.870	0.774	0.781	/	0.829	/	/	0.699	0.077
[15]	40	0.870	/	/	0.820	/	/	0.780	/	/	/	/
[16]	27	0.824	/	/	0.745	/	/	0.649	/	/	/	/

**Table 4:** Physical Meaning of Molecular Descriptors Selected in Models 1 and 2.

Name	Physical Meaning
AATS7Z	Averaged Moreau-Broto autocorrelation of lag 7 weighted by atomic number.
GATS2are	Geary coefficient of lag 2 weighted by Allred-Rocow electronegativity.
GATS5c	Geary coefficient of lag 5 weighted by Gasteiger charge.
PEOE_VSA10	MOE Charge VSA Descriptor 10 (0.10≤x<0.15 partial charge range).
PEOE_VSA6	MOE Charge VSA Descriptor 6 (−0.10≤x<−0.05 partial charge range).
SMR_VSA6	MOE MR VSA Descriptor 6 (2.75≤x<3.05 partial molar refractivity range).
EState_VSA9	EState VSA Descriptor 9 (4.69≤x<9.17 EState contribution range).
EState_VSA4	EState VSA Descriptor 4 (0.72≤x<1.17 EState contribution range).
NaaaC	Number of C atoms shared by an aromatic ring.
NssssC	Count of atom-type E-state: >C< (quaternary carbon).
naHRing	Aromatic ring count containing heteroatoms.
n10FaRing	Count of 10-membered aromatic fused rings.
SssNH	Sum of –NH– electrotopological-states.
StsC	Sum of ≡C− EStates (acetylene/alkyne-type carbon).

**Table 5:** Values of Descriptors of Model 1 for Selected Molecules from the CHEMBL Database.

**Molecule 1 (${\mathrm{pIC}}_{50}=7.3$)**	**Molecule 2 (${\mathrm{pIC}}_{50}=7.2$)**	**Molecule 3 (${\mathrm{pIC}}_{50}=6.4$)**
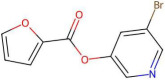	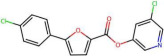	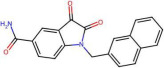
**Descriptor Values**		
AATS7Z = 36.7, EState_VSA9 = 9.2	AATS7Z = 30.0, EState_VSA9 = 32.4	AATS7Z = 19.7, EState_VSA9 = 5.7
PEOE_VSA10 = 0.0, GATS5c = 1.3	PEOE_VSA10 = 5.8, GATS5c = 1.4	PEOE_VSA10 = 0.0, GATS5c = 1.1
PEOE_VSA6 = 0.0, GATS2are = 1.0	PEOE_VSA6 = 0.0, GATS2are = 0.8	PEOE_VSA6 = 36.4, GATS2are = 0.5
EState_VSA4 = 4.4, n10FaRing = 0	EState_VSA4 = 5.6, n10FaRing = 0	EState_VSA4 = 5.6, n10FaRing = 1
SMR_VSA6 = 0.0, SssNH = 0.0	SMR_VSA6 = 23.2, SssNH = 0.0	SMR_VSA6 = 16.3, SssNH = 0.0
NaaaC = 0, naHRing = 2	NaaaC = 0, naHRing = 2	NaaaC = 2, naHRing = 0
StsC = 0.0, NssssC = 0	StsC = 0.0, NssssC = 0	StsC = 0.0, NssssC = 0
**Molecule 4** (${\mathrm{pIC}}_{50}=4.7$)	**Molecule 5** (${\mathrm{pIC}}_{50}=3.7$)	**Molecule 6** (${\mathrm{pIC}}_{50}=3.3$)
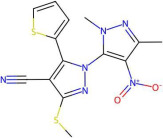	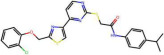	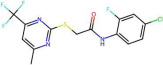
**Descriptor Values**		
AATS7Z = 27.4, EState_VSA9 = 0.0	AATS7Z = 24.2, EState_VSA9 = 16.3	AATS7Z = 25.7, EState_VSA9 = 11.6
PEOE_VSA10 = 28.1, GATS5c = 1.1	PEOE_VSA10 = 23.1, GATS5c = 0.8	PEOE_VSA10 = 11.5, GATS5c = 0.6
PEOE_VSA6 = 6.1, GATS2are = 0.6	PEOE_VSA6 = 61.5, GATS2are = 0.8	PEOE_VSA6 = 23.4, GATS2are = 0.5
EState_VSA4 = 4.9, n10FaRing = 0	EState_VSA4 = 16.4, n10FaRing = 0	EState_VSA4 = 23.9, n10FaRing = 0
SMR_VSA6 = 6.3, SssNH = 0.0	SMR_VSA6 = 11.1, SssNH = −2.9	SMR_VSA6 = 11.1, SssNH = −2.3
NaaaC = 0, naHRing = 3	NaaaC = 0, naHRing = 2	NaaaC = 0, naHRing = 1
StsC = 2.2, NssssC = 0	StsC = 0.0, NssssC = 0	StsC = 0.0, NssssC = 1

## Data Availability

The original contributions presented in this study are included in the article and Supplementary Material. Further inquiries can be directed to the corresponding author.

## Supplementary Materials

The following supporting information can be downloaded at: https://www.mdpi.com/article/10.3390/ph19030498/s1, File S1: Canonical smiles and corresponding pIC_50_ values for the molecules from the ChEMBL dataset; File S2: Canonical smiles and corresponding pIC_50_ values for the molecules in the external dataset.

## Appendix A. Experimental

In the following report, we present the best OLS models obtained using RFE, SequentialFeatureSelector, and SelectKBest feature selection algorithms (Equations (A1), (A2) and (A3), respectively)

(A1) $$\begin{array}{cc} {\mathrm{pIC}}_{50}= & 1.76\cdot 10^{-3}\cdot \;\mathrm{AATS}6\mathrm{dv}\;+0.69\cdot \mathrm{AATS}4d+1.79\cdot \mathrm{GATS}5c+2.64\cdot \mathrm{GATS}1p\\ \; & -2.81\cdot \;\mathrm{GATS}2p-2.28\cdot \mathrm{GATS}3p-0.29\cdot C1\mathrm{SP}3+0.32\cdot \mathrm{NdssC}\\ \; & +0.46\cdot \;\mathrm{naHRing}+1.17\cdot \mathrm{nFaRing}-0.93\cdot \mathrm{GGI}3-5.52\cdot \mathrm{JGI}1+3.16 \end{array}$$

(A2) $$\begin{array}{cc} {\mathrm{pIC}}_{50}= & -1.22\cdot \;\mathrm{AATS}2d\;-2.24\cdot 10^{-5}\cdot \;\mathrm{AATS}6v\;+8.75\cdot 10^{-3}\cdot \;\mathrm{AATS}7v\;\\ \; & -1.44\cdot 10^{-2}\cdot \;\mathrm{ATSC}0i\;+9.92\cdot 10^{-3}\cdot \;\mathrm{ATSC}2i\;-99.30\cdot \;\mathrm{AATSC}6c\;\\ \; & +0.17\cdot \;\mathrm{AATSC}8\mathrm{dv}\;-0.59\cdot \;\mathrm{AATSC}6i\;+1.90\cdot \;\mathrm{GATS}1p\;\\ \; & -0.49\cdot \;\mathrm{IC}1\;+0.90\cdot \;n10\mathrm{FaRing}\;-10.08\mathrm{JGI}2+1.51 \end{array}$$

(A3) $$\begin{array}{cc} {\mathrm{pIC}}_{50}= & -0.49\cdot \;\mathrm{AATS}2d\;+1.46\cdot \;\mathrm{AATS}5d\;+0.22\cdot \;\mathrm{AATS}6d\;-1.32\cdot 10^{-3}\cdot \;\mathrm{AATS}7m\;\\ \; & -2.81\cdot 10^{-3}\cdot \;\mathrm{AATS}5v-4.58\cdot 10^{-3}\cdot \;\mathrm{AATS}6v\;-4.34\cdot 10^{-2}\cdot \;\mathrm{SMR}\_\mathrm{VSA}6\\ \; & +5.61\cdot 10^{-3}\cdot \;\mathrm{AATS}7v+0.71\cdot \;\mathrm{AATS}8p\;+\;16.90\cdot \;\mathrm{AATSC}3c\;\\ \; & +1.08\cdot 10^{2}\cdot \;\mathrm{AATSC}6c\;-1.93\cdot \mathrm{FCSP}3-0.10\cdot \;\mathrm{nHBDon}\;+4.31 \end{array}$$

Then, we present the results of their internal fitting (Table A1) and predictive performance (Table A2) parameters, as well as results for internal fitting (Table A3) and predictive performance (Table A4) parameters for models obtained using several linear regression methods combined with descriptors from Models 1, 2, and 3 given in the manuscript. “B Ridge” stands for Bayesian Ridge Regression, while “LSVR” is Linear Support Vector Regression.

**Table A1:** Internal Statistical Fitting Parameters of the QSAR Models.

Model	$R^{2}$	$R_{\mathrm{adj}}^{2}$	${\mathrm{RMSE}}_{\mathrm{tr}}$	${\mathrm{CCC}}_{\mathrm{tr}}$	F	$Q_{\mathrm{LOO}}^{2}$	${\mathrm{RMSE}}_{\mathrm{cv}}$	${\mathrm{CCC}}_{\mathrm{cv}}$	$Q_{\mathrm{LMO}}^{2}$	$R_{\mathrm{Yscr}}^{2}$	$Q_{\mathrm{Yscr}}^{2}$
S1	0.859	0.828	0.325	0.924	28	0.779	0.408	0.879	0.719	0.157	−0.309
S2	0.853	0.823	0.331	0.920	28	0.778	0.459	0.872	0.711	0.171	−0.288
S3	0.790	0.765	0.410	0.874	11	0.710	0.482	0.865	0.650	0.201	−0.277

**Table A2:** Predictive Performance Parameters of the QSAR Models on the External Test Set.

Model	$R_{\mathrm{ext}}^{2}$	${\mathrm{RMSE}}_{\mathrm{ext}}$	$Q_{F1}^{2}$	$Q_{F2}^{2}$	$Q_{F3}^{2}$	${\mathrm{CCC}}_{\mathrm{ext}}$	${\mathrm{MAE}}_{\mathrm{ext}}$	$r_{m}^{2}$	$\Delta r_{m}^{2}$	k	$k^{\prime }$	$\frac{R_{\mathrm{ext}}^{2}-R_{0}^{2}}{R_{\mathrm{ext}}^{2}}$	$\frac{R_{\mathrm{ext}}^{2}-R_{0}^{\prime 2}}{R_{\mathrm{ext}}^{2}}$
S1	0.855	0.439	0.853	0.853	0.748	0.905	0.364	0.789	0.128	0.985	1.010	0.013	0.001
S2	0.846	0.446	0.846	0.845	0.722	0.985	0.377	0.771	0.133	1.005	0.985	0.013	0.127
S3	0.782	0.504	0.724	0.724	0.718	0.877	0.409	0.693	0.167	1.006	0.985	0.005	0.084

**Table A3:** Internal Statistical Fitting Parameters for Various Regression Models.

Model	$R^{2}$	$R_{\mathrm{adj}}^{2}$	${\mathrm{RMSE}}_{\mathrm{tr}}$	${\mathrm{CCC}}_{\mathrm{tr}}$	F	$Q_{\mathrm{LOO}}^{2}$	${\mathrm{RMSE}}_{\mathrm{cv}}$	${\mathrm{CCC}}_{\mathrm{cv}}$	$Q_{\mathrm{LMO}}^{2}$	$R_{\mathrm{Yscr}}^{2}$	$Q_{\mathrm{Yscr}}^{2}$
Ridge 1	0.867	0.811	0.359	0.913	22	0.806	0.454	0.876	0.721	0.198	−0.364
LassoLars 1	0.869	0.813	0.357	0.916	23	0.806	0.454	0.876	0.721	0.198	−0.364
B Ridge 1	0.863	0.807	0.363	0.909	20	0.806	0.454	0.876	0.721	0.198	−0.364
ARD 1	0.861	0.804	0.365	0.908	21	0.806	0.454	0.876	0.721	0.198	−0.364
Lasso 1	0.864	0.807	0.362	0.909	20	0.806	0.444	0.876	0.721	0.198	−0.364
LSVR 1	0.820	0.752	0.408	0.880	16	0.806	0.444	0.876	0.656	0.198	−0.364
Ridge 2	0.853	0.810	0.374	0.904	29	0.766	0.446	0.878	0.680	0.147	−0.264
LassoLars 2	0.855	0.812	0.372	0.908	31	0.766	0.446	0.878	0.680	0.147	−0.264
B Ridge 2	0.852	0.809	0.375	0.903	28	0.776	0.446	0.878	0.680	0.147	−0.264
ARD 2	0.852	0.809	0.375	0.903	28	0.766	0.446	0.878	0.680	0.147	−0.264
Lasso 2	0.851	0.807	0.377	0.900	27	0.776	0.436	0.878	0.680	0.147	−0.264
LSVR 2	0.831	0.784	0.397	0.886	24	0.776	0.436	0.878	0.749	0.147	−0.264
Ridge 3	0.828	0.774	0.389	0.896	19	0.732	0.458	0.854	0.687	0.184	−0.364
LassoLars 3	0.840	0.787	0.386	0.901	20	0.722	0.458	0.854	0.687	0.184	−0.364
B Ridge 3	0.837	0.783	0.390	0.894	18	0.722	0.458	0.854	0.687	0.184	−0.364
ARD 3	0.827	0.770	0.400	0.889	17	0.722	0.458	0.854	0.687	0.184	−0.364
Lasso 3	0.830	0.775	0.397	0.889	17	0.722	0.448	0.854	0.687	0.184	−0.364
LSVR 3	0.778	0.710	0.448	0.840	10	0.722	0.448	0.854	0.659	0.184	−0.364

**Table A4:** Predictive Performance Parameters of Various Regression Models on the External Test Set.

Model	$R_{\mathrm{ext}}^{2}$	${\mathrm{RMSE}}_{\mathrm{ext}}$	$Q_{F1}^{2}$	$Q_{F2}^{2}$	$Q_{F3}^{2}$	${\mathrm{CCC}}_{\mathrm{ext}}$	${\mathrm{MAE}}_{\mathrm{ext}}$	$r_{m}^{2}$	$\Delta r_{m}^{2}$	k	$k^{\prime }$	$\frac{R_{\mathrm{ext}}^{2}-R_{0}^{2}}{R_{\mathrm{ext}}^{2}}$	$\frac{R_{\mathrm{ext}}^{2}-R_{0}^{\prime 2}}{R_{\mathrm{ext}}^{2}}$
Ridge 1	0.861	0.465	0.859	0.858	0.750	0.907	0.402	0.782	0.148	1.006	0.984	0.000	0.035
LassoLars 1	0.865	0.461	0.863	0.859	0.754	0.911	0.394	0.788	0.141	1.004	0.986	0.003	0.024
B Ridge 1	0.858	0.469	0.855	0.855	0.755	0.903	0.407	0.774	0.153	1.007	0.982	0.000	0.056
ARD 1	0.862	0.466	0.857	0.857	0.759	0.906	0.403	0.783	0.150	1.010	0.980	0.000	0.048
Lasso 1	0.860	0.468	0.856	0.855	0.756	0.904	0.406	0.779	0.151	1.009	0.981	0.000	0.054
LSVR 1	0.827	0.557	0.787	0.786	0.646	0.873	0.434	0.738	0.148	1.050	0.949	0.011	0.033
Ridge 2	0.844	0.495	0.838	0.837	0.739	0.901	0.410	0.765	0.143	1.013	0.987	0.002	0.048
LassoLars 2	0.846	0.494	0.839	0.838	0.741	0.904	0.405	0.769	0.146	1.011	0.989	0.004	0.046
B Ridge 2	0.844	0.496	0.837	0.837	0.738	0.900	0.411	0.764	0.156	1.013	0.987	0.004	0.061
ARD 2	0.845	0.494	0.839	0.838	0.741	0.902	0.407	0.767	0.143	1.013	0.987	0.004	0.048
Lasso 2	0.843	0.498	0.836	0.835	0.736	0.889	0.412	0.758	0.150	1.014	0.986	0.004	0.055
LSVR 2	0.810	0.536	0.785	0.784	0.655	0.872	0.450	0.720	0.160	1.034	0.965	0.006	0.052
Ridge 3	0.820	0.502	0.813	0.812	0.722	0.891	0.456	0.662	0.175	1.020	0.989	0.020	0.107
LassoLars 3	0.839	0.499	0.825	0.824	0.725	0.895	0.451	0.688	0.187	1.026	0.992	0.015	0.084
B Ridge 3	0.831	0.503	0.822	0.821	0.720	0.889	0.457	0.656	0.187	1.020	0.988	0.021	0.113
ARD 3	0.819	0.526	0.805	0.805	0.704	0.882	0.480	0.664	0.193	1.026	0.982	0.014	0.099
Lasso 3	0.822	0.515	0.814	0.813	0.707	0.884	0.470	0.647	0.193	1.023	0.984	0.021	0.120
LSVR 3	0.733	0.564	0.726	0.725	0.656	0.845	0.494	0.519	0.260	1.029	0.967	0.062	0.306
